# Can a Total Knee System Providing 1 mm Increment of Polyethylene Insert Thickness Offer a Clinical Benefit?

**DOI:** 10.3390/medicina60020322

**Published:** 2024-02-14

**Authors:** Dhong-Won Lee, Hyuk-Jin Jang, Man-Soo Kim, Keun-Young Choi, Sung-An Hong, Yong In

**Affiliations:** 1 Department of Orthopaedic Surgery, Konkuk University Medical Center, Konkuk University School of Medicine, Seoul 05030, Republic of Korea; osdoctorknee@kuh.ac.kr; 2 Department of Orthopaedic Surgery, Seoul St. Mary’s Hospital, College of Medicine, The Catholic University of Korea, 222, Banpo-daero, Seocho-gu, Seoul 06591, Republic of Korea; drjack5546@daum.net (H.-J.J.); kms3779@naver.com (M.-S.K.); heaxagon@hanmail.net (K.-Y.C.); ydeepinyou@cmcnu.or.kr (S.-A.H.)

**Keywords:** total knee arthroplasty, polyethylene thickness, 1 mm increment, flexion contracture, recurvatum, randomized controlled trial

## Abstract

*Background and Objectives*: The purpose of this study was to compare clinical outcomes and polyethylene (PE) insert thickness between total knee arthroplasty (TKA) systems providing 1 mm and 2 mm increments. *Materials and Methods*: In this randomized controlled trial, 50 patients (100 knees) undergoing same-day or staggered bilateral TKA were randomized to receive a TKA system providing 1 mm increments in one knee (1 mm group) and a TKA system providing 2 mm increments in the other knee (2 mm group). At 2 years postoperatively, Knee Society Score (KSS), Western Ontario and McMaster Universities Osteoarthritis Index (WOMAC) score, Forgotten Joint Score (FJS), range of motion (ROM), and insert thicknesses were compared between the groups. *Results*: A total of 47 patients (94 knees) participated in follow-up analysis. In each group, patient-reported outcomes improved significantly after TKA (all, *p* < 0.05). There were no significant differences in patient-reported outcomes. The mean ROM was not significantly different between groups at preoperative and 2-year points. The rate of postoperative flexion contracture ≥ 5° was 2.1% and 4.3%, and the rate of postoperative recurvatum ≥ 5° was 4.3% and 2.1% in the 1 mm group and 2 mm, respectively (all, *p* = 1.000). Mean insert thickness was significantly thinner in the 1 mm group than the 2 mm group (*p* = 0.001). The usage rate of a thick insert (≥14 mm) was 12.7% and 38.3% in the 1 mm group and 2 mm group (*p* = 0.005). *Conclusions*: The use of a TKA system providing 1 mm PE insert thickness increments offered no clinical benefit in terms of patient reported outcomes over systems with 2 mm increments at 2 years of follow-up. However, the TKA system with 1 mm increments showed significantly thinner PE insert usage. As a theoretical advantage of 1 mm increments has yet to be proven, the mid- to long-term effects of thinner PE insert usage must be determined.

## 1. Introduction

Total knee arthroplasty (TKA) generally provides effective pain relief and improved function for patients with advanced symptomatic osteoarthritis. However, some patients express dissatisfaction with the outcome [1,2,3]. It is important to achieve optimal soft tissue balance to elicit satisfactory kinematics after TKA [4,5,6,7,8,9,10]. This balance is judged by evaluating the medial and lateral symmetry of the flexion and extension gaps, as well as the effects of implants of different sizes [5,6,7,9,10]. Failure to achieve soft tissue balance may lead to complications such as restricted range of motion (ROM), pain, and instability, possibly necessitating revision surgery [11,12,13]. Researchers have proposed various methods to achieve soft tissue balance, but the most accurate method has not yet been established [14,15,16,17,18]. The selection of an appropriate polyethylene (PE) insert thickness is one such approach based on the assessment of soft tissue tension and joint stability during TKA [19,20,21,22]. Trial inserts of different thicknesses are available to the surgeon to test the soft tissue balance and joint stability [23]. The surgeon can place different thicknesses of trial inserts and assess the joint kinematics to determine the optimal thickness that provides balanced soft tissue tension throughout the range of motion. Based on the intraoperative assessment and trial insert testing, the surgeon selects the final PE insert thickness. The chosen thickness should achieve optimal soft tissue balance, stability, and kinematics of the knee joint. Therefore, it is reasonable to provide implants with smaller PE insert increments [24]. Traditionally, PE inserts have been available in thicknesses starting from 9 mm or 10 mm, increasing in 2 mm increments [25,26]. For thicknesses of 13 mm or 14 mm and greater, the inserts often increase by 3 mm. Recently, prostheses have been designed to allow PE insert thickness increments of 1 mm [25,26]. With a variable range of PE insert thicknesses, surgeons can more precisely achieve soft tissue balance, ensuring knee stability and more easily reducing extension loss [24,25].

To demonstrate the superiority of 1 mm increments over 2 mm increments in terms of PE insert thicknesses, it must be established that the 1 mm increments provide sufficient stability while avoiding issues such as flexion contracture or recurvatum of the knee [1]. However, few studies have reported on the effect of PE insert thickness to improve clinical outcomes and implant survival. Several reports have compared clinical outcomes and implant survival between thick and thin PE inserts, but they have presented conflicting results [27,28,29,30]. Moreover, the thick PE insert used in previous studies was limited to approximately 15–16 mm, allowing its use in only a subset of patients [28,29]. Consequently, the pool of candidates for a thick PE insert was relatively small, and the difference in thickness between PE inserts was substantial [28,29]. Furthermore, previous studies lacked control over patient factors, limiting the interpretation and clinical applicability of the findings.

This prospective randomized study was performed to compare clinical outcomes and PE insert thicknesses between TKA systems providing 1 mm and 2 mm PE insert increments. We hypothesized that the TKA system with a 1 mm increment of PE insert thickness would show better clinical outcomes and would lead to use of a thinner PE insert.

## 2. Materials and Methods

**Figure 1:**
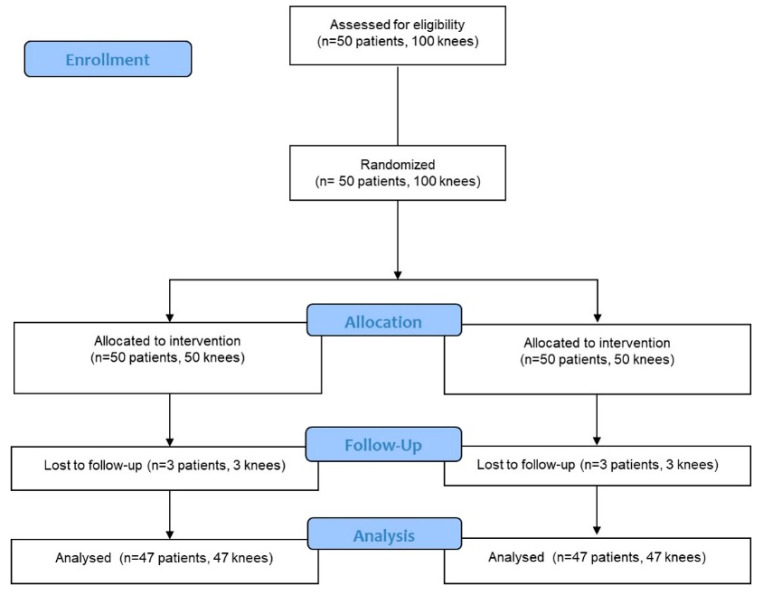
Consolidated standards of reporting trials flow diagram.

This prospective randomized controlled trial (RCT) was approved by the Institutional Review Board of our hospital (KC20DISI0965) and was designed as a single-institution parallel-group study with balanced randomization. This study was registered at ClinicalTrials.gov (NCT04687462). All patients were educated regarding the study requirements and provided informed consent. Patients undergoing bilateral TKA either on the same-day or within one week from March 2020 to December 2021 were consecutively enrolled. Patients were excluded if they had inflammatory or secondary arthritis, a history of high tibial osteotomy, flexion contracture greater than 25° or further flexion less than 90°, or if they refused to participate in the study. A total of 50 patients (100 knees) were enrolled. One knee was randomly assigned to a posterior-stabilized (PS) TKA system providing a 1 mm increment of PE insert thickness (Exult Knee System, Corentec Co., Ltd., Seoul, Republic of Korea) (1 mm group) and the other knee was assigned to a PS TKA system providing a 2 mm increment of PE insert thickness (LOSPA Knee System, Corentec Co., Ltd., Seoul, Republic of Korea) (2 mm group) using a computer-generated randomization table created prior to surgery (Figure 1). A total of 29 patients underwent same-day bilateral TKA and 21 patients underwent staggered TKA with a one-week interval. The implants shared a single-axis design for articular stability with flexion and improved ROM, a patella-friendly design (lateralized curved trochlear groove), and high flexion. The Exult Knee System has 1 mm PE insert increments from 9 to 14 mm and 2 mm increments from 16 to 18 mm, while the Lospa Knee System has 2 mm PE increments from 10 to 18 mm. During the study period, the patients and the investigators collecting data were blinded to the TKA system group allocation.

**Figure 2:**
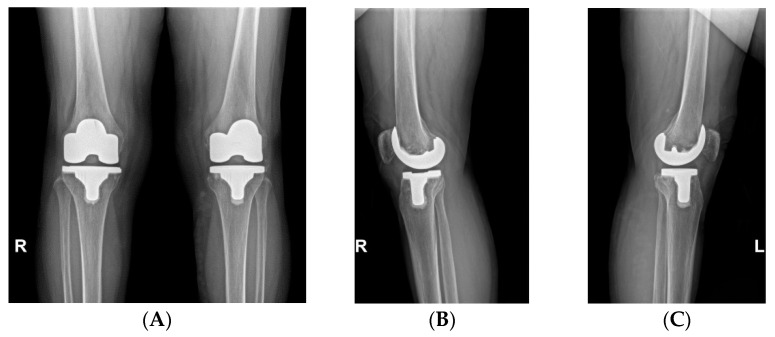
Postoperative radiographs. (**A**) Anteroposterior radiograph of a patient who underwent a right total knee arthroplasty (TKA) providing 1 mm increment of PE insert thickness (Exult) and a left TKA providing 2 mm increment (LOSPA). (**B**) Lateral radiograph of a right TKA providing 1 mm increment. (**C**) Lateral radiograph of a left TKA providing 2 mm increment.

All TKAs were performed by a senior surgeon (Y. I.) who used a subvastus approach with patellar sliding and a measured resection technique. The medial release was performed in three stages based on the degree of medial tightness. The first step was deep medial collateral ligament (MCL) release using subperiosteal elevator from the menisco-capsular junction. If there was residual medial tightness after tibial and femoral bone cutting, semimembranosus release was performed. Finally, superficial MCL release using the pie-crust method with an 18-gauge needle was performed if there was residual medial tightness [16]. To achieve equal extension and flexion gaps when inserting trial components, appropriate soft tissue balancing using a gapper device (B. Braun, Melsungen, Germany) was conducted. The force applied to the gapper device during flexion and extension was considered balanced when it was equal, and the soft tissue balance was deemed appropriate when it neither felt too tight to move nor too loose to move freely. Ultimately, the soft tissue balancing determination was that after performing bone resection and applying balanced forces to the knee, the gap should remain equal on the medial and lateral sides without forming a trapezoid shape. During the insertion of the trial components, it was ensured that, during flexion and extension of the knee joint, neither side was overly tensioned nor excessively lax. Before cementing, trial components were inserted. The PE trial increments between Exult and Lospa systems were different. In the Exult system, it was possible to achieve a soft tissue balance with a 1 mm increment, while in the Lospa system, soft tissue balance needs to be adjusted with a 2 mm interval. After achieving equal flexion and extension gaps, the trial PE insert size was determined in extension to prevent the occurrence of recurvatum or flexion contracture. Both the senior surgeon’s and assistants’ consensus was sought in determining the thickness, and a slight extension tightness was tolerated. The femoral and tibial components were cemented. PE insert thickness was finally selected based on the absence of flexion contracture or recurvatum. No patellae were resurfaced in any case (Figure 2).

All patients received the same peri-operative multimodal pain control including pre-emptive oral analgesics, postoperative intravenous patient-controlled analgesia pump (1 mL of a 100 mL solution containing 2000 μg of fentanyl) and oral analgesics, and anti-inflammatory medication (10 mg of oxycodone every 12 h for 1 week along with 200 mg of celecoxib, 37.5 mg of tramadol, and 650 mg of acetaminophen every 12 h for 6 weeks) [31,32]. All patients began tolerable ROM exercise and were encouraged to walk using a walker on the first postoperative day.

Clinical assessments were performed preoperatively and postoperatively at 6 months, 1 year, and annually thereafter. A 60 cm goniometer was used to measure the ROM in a supine position. In addition, PE insert thickness was compared between the groups. The primary outcome was Knee Society Score (KSS). The secondary outcomes were Western Ontario and McMaster Universities Osteoarthritis Index (WOMAC) score, Forgotten Joint Score (FJS), and ROM. Radiological assessment included components positions, lower limb alignments, posterior condylar offset (PCO), and PCO ratio of both knees.

Statistical analysis was performed using SPSS software (IBM SPSS Statistics 21; IBM Corp, Somers, NY, USA) with statistical significance set at *p* < 0.05. A priori power analysis revealed that the detection of a standardized difference of 1 in KSS satisfaction between the two groups required a sample size of 48 to achieve a power of 0.80 (type II error) and an alpha of 0.01 (type 1 error) [33]. Considering a 10% dropout rate, 47 knees per group were adequate. Student’s *t* test was used to compare continuous variables between both groups. Preoperative and postoperative parametric or non-parametric variables were compared using the paired *t*-test or Wilcoxon signed-rank test. Categorical variables were compared with Chi-square tests.

## 3. Results

At 2 years postoperatively, 47 patients (94 knees) were followed up. There was 1 male and 46 females. Patients’ mean age was 68.7 ± 5.8 years. Their mean body mass index was 27.3 ± 4.1 kg/m^2^. In each group, the KSS, WOMAC score, and FJS improved significantly after TKA (all, *p* < 0.05). The mean total KSS at 2 years was 139.1 ± 24.8 in the 1 mm group and 136.3 ± 28.5 in the 2 mm group, with no significant difference (*p* = 0.613). The mean WOMAC score was 21.5 ± 6.1 in the 1 mm group and 23.0 ± 5.7 in the 2 mm group, with no significant difference (*p* = 0.221). The mean postoperative FJS was 58.5 ± 13.4 in the 1 mm group and 55.9 ± 14.1 in the 2 mm group, with no significant difference (*p* = 0.362) (Table 1).

The mean ROM was not significantly different between the groups at preoperative and 2-year points. The ratio of preoperative flexion contracture ≥ 5° was 53.2% and 51.1% in the 1 mm group and 2 mm group (*p* = 0.836), and postoperative flexion contracture ≥ 5° was 2.1% and 4.3%, respectively (*p* = 1.000). The rate of preoperative recurvatum ≥ 5° was 0% in both groups, and the rate of postoperative recurvatum ≥ 5° was 4.3% and 2.1%, respectively (*p* = 1.000). On X-rays, there were no significant differences in PCO, PCO ratio, and hip–knee–ankle axis angle between the two groups at either the preoperative or 2-year point (Table 2).

**Figure 3:**
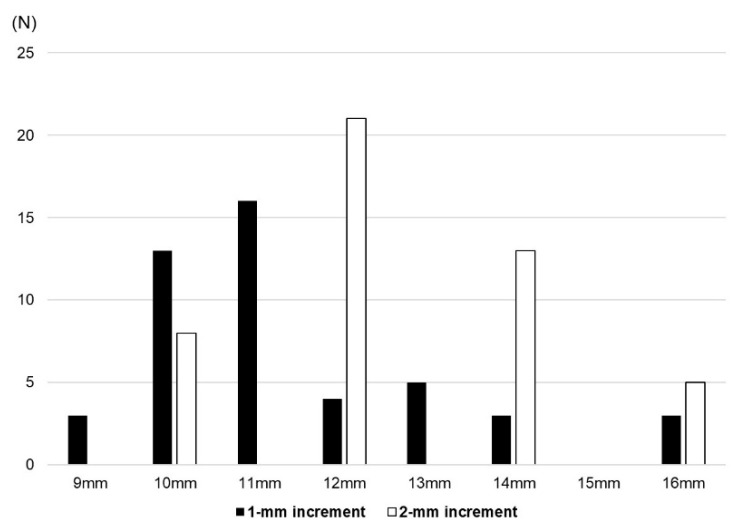
Distribution of polyethylene insert thickness in the two groups.

**Figure 4:**
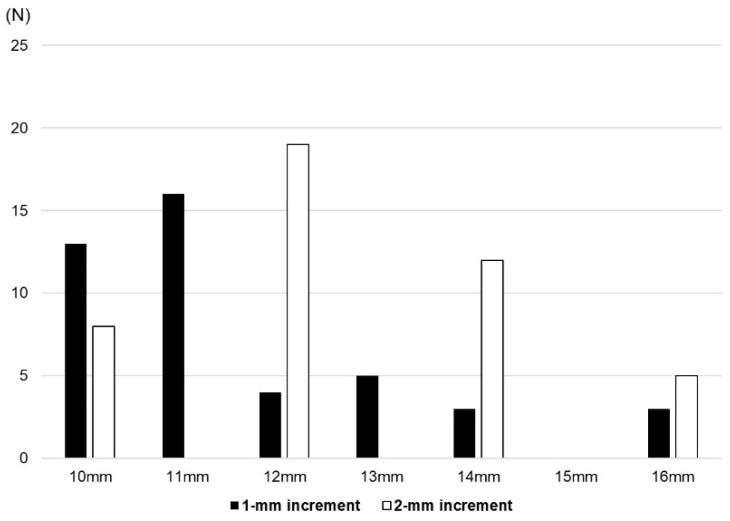
Distribution of polyethylene insert thickness in the two groups after excluding three patients who used a 9 mm insert.

The mean PE insert thickness was significantly thinner in the 1 mm group than the 2 mm group (11.5 ± 1.8 mm vs. 12.8 ± 1.6 mm, *p* = 0.001). The minimum PE insert thickness for Exult is 9 mm and that for Lospa is 10 mm (Figure 3). Even when excluding three patients who used a 9 mm PE insert of the Exult knee system on one knee, the mean PE thickness of the 1 mm group was significantly thinner (11.6 ± 1.7 mm vs. 12.7 ± 1.8 mm, *p* = 0.008) (Figure 4). The rate of thick PE insert usage (≥14 mm) was 12.7% and 38.3% in the 1 mm group and 2 mm group, respectively (*p* = 0.005) (Table 3).

## 4. Discussion

The most important finding of this study was that clinical outcomes were improved after TKA regardless of thickness increment, and there were no significant differences in clinical outcomes between the two groups at short-term follow-up. However, the TKA system with 1 mm increments used significantly thinner PE inserts with a lower rate of thick PE insert (≥14 mm) usage than the system with 2 mm increments.

Theoretically, a 1 mm thickness adjustment TKA system could provide a more precise gap balance, potentially improving clinical outcomes. However, few studies of a TKA system using the 1 mm thickness adjustment PE insert rather than the existing 2 mm thickness adjustment have been conducted. Lanting et al. [24] reported that 1 mm increments did not induce flexion contracture, but 2 mm increments caused flexion contracture in 66% of TKAs with appropriate gap balance due to its larger effect on the extension angle rather than the flexion angle. The authors demonstrated that a 1 mm adjustment produced significant changes in soft tissue balance, and a minimum 2 mm interval between PE insert thicknesses may not be sufficient to optimize soft tissue balance using computer-based navigation. Kishimura et al. [34] also suggested 1 mm increments to minimize gap difference and to resolve flexion contracture. They found that gap differences negatively correlated with the extension angle at TKA, and this correlation was maintained at 2 years using a specially designed tensor device. Song et al. [26] reported that the PE insert thickness of a TKA system providing 1 mm increments was significantly thinner than that of the system with 2 mm increments, and 1 mm increments could decrease the posterior tibial slope and the incidence of excessive posterior tibial slope in cruciate-retaining (CR) TKA. The authors found no significant differences in clinical score and range of motion between 1 mm increment and 2 mm increment CR TKA systems. In the current study, a PS TKA system providing 1 mm increments showed significantly thinner PE thickness than a PS TKA system with 2 mm increments; however, there were no significant differences in patient-reported outcomes and the incidence of flexion contracture or recurvatum. These findings imply that appropriate implant design and sound surgical technique are more critical than PE insert thickness. However, during extension gap establishment, flexion contracture may occur due to a 1 mm difference, underscoring the significance of precision in this aspect [35].

In previous studies, a thick PE insert was defined as 15–16 mm or more; more recently, it has been defined as 13–14 mm or more [27,28,30]. In the present study, when comparing the two groups with the threshold of thick PE as 14 mm, there was a significant difference in the usage rate of thick PE inserts between groups. However, there was no difference with the threshold of thick PE as 15 mm [20,30]. We found that the TKA system providing 1 mm increments showed a significantly lower rate of thick PE insert usage than the system providing 2 mm increments. Berend et al. [28] reported that thicker bearings (16–20 mm) were significantly associated with higher failure rates than thinner bearings (8–14 mm) at mid- to long-term follow-ups. The authors concluded that the use of a thicker PE insert itself did not cause failure, but that factors leading to the use of a thicker PE insert, such as deeper tibial resection and excessive ligament release necessitated by severe deformity, were associated with higher failure rates. Rajamaki et al. [27] revealed that the thick PE insert group (≥13 mm) showed an increased revision risk compared with the standard PE insert group (<13 mm) in both short- and long-term follow-up. They suggest that if a thick PE insert is required to achieve ligamentous stability and gap balance even after standard tibial cutting (9–10 mm) and soft tissue balancing, surgeons should consider using a more constrained TKA design. In other words, the reason for the higher failure rates observed after using thicker PE inserts in the two previous studies was not because of the thicker PE insert itself but rather to the factors leading to its use. In the present study, there were no significant differences in preoperative hip–knee–ankle axis angle, PCO, PCO ratio, ROM, and flexion contracture or recurvatum between TKA systems providing 1 mm increments and 2 mm increments. The reason for the difference in mean PE thickness under similar preoperative conditions was the ability to use 11 mm and 13 mm PE inserts with the TKA system providing 1 mm increments. For instance, if the soft tissue tension is lax at 12 mm and tight at 14 mm, a thickness of 13 mm can help avoid the use of 14 mm, reducing the risk of flexion contracture. Moreover, after performing standard tibial cutting (9–10 mm from the lateral plateau in varus knee), a PE insert with a thickness of 11–12 mm is sometimes required in clinical practice. For example, even if the preoperative deformity is similar and tibial cutting is performed with the same thickness, some patients may require a 10 mm PE insert, while others may require an 11 mm PE insert. In patients with severe varus or valgus deformities, a thicker PE insert might be required after soft tissue balancing compared with inserts suitable for patients with milder deformities. However, considering that among knees with similar deformities, patients can exhibit subtle differences, the use of 1 mm increments in PE may help contribute to personalized surgical treatment.

To the best of our knowledge, this study is the first randomized controlled trial to investigate the usefulness of 1 mm increments of PE insert in a PS TKA system. We tried to control surgery-specific and patient-specific factors, and only patients with same-day or 1-week staggered bilateral TKA (one side with 1 mm increments and the other with 2 mm increments) performed by an experienced surgeon were included. We found possible benefits of using a thinner PE insert in the 1 mm group even though there were no differences in clinical outcomes. While the benefits of use of thinner PE insert have not been demonstrated at short-term follow-up, further observation is required to assess its clinical advantage at mid- to long-term follow-up.

This study has several limitations. First, the 2-year follow-up period was relatively short to determine clinical outcomes and the effects of thinner PE inserts. Mid- and long-term follow-up is needed to reveal the benefits of the TKA system providing 1 mm increments. Second, the implant products used in the two groups were different. However, the LOSPA and EXULT systems were manufactured by the same company and share key features such as a single-axis, patella-friendly, and high-flexion design. Thus, we anticipate no significant outcome differences based on the implant. Third, the intraoperative soft tissue balancing technique could have affected the flexion and extension gaps. However, the surgery was performed by one senior surgeon with more than 20 years of experience, and soft tissue balancing was performed according to the same principles. In our practice, a gapper device and trial components were used to check the gap balance. However, it is essential to note the limitation due to the absence of the use of a digitalized gap evaluation device such as navigation or robotic system.

## 5. Conclusions

The use of a TKA system providing 1 mm PE insert thickness increments offered no clinical benefit in terms of patient-reported outcomes over systems with 2 mm increments at 2 years of follow-up. However, the TKA system with 1 mm increments showed significantly thinner PE insert usage. As a theoretical advantage of 1 mm increments has yet to be proven, the mid- to long-term effects of thinner PE insert usage must be determined.

## Figures and Tables

**Table 1:** Comparison of clinical outcomes between the two groups at preoperative and 2-year postoperative points.

	1-mm Group (N = 47)	2-mm Group (N = 47)	*p* Value
Preoperative KSS, total	99.5 ± 36.4	100.9 ± 38.0	0.856
Postoperative KSS, total	139.1 ± 24.8	136.3 ± 28.5	0.613
Preoperative KSS-Pain	14.6 ± 8.2	15.7 ± 8.9	0.535
Postoperative KSS-Pain	42.6 ± 12.9	41.6 ± 13.3	0.712
Preoperative KSS-Function	84.5 ± 24.1	85.5 ± 21.8	0.833
Postoperative KSS-Function	96.7 ± 25.0	94.0 ± 22.4	0.583
Preoperative WOMAC score	48.2 ± 10.0	46.0 ± 12.6	0.351
Postoperative WOMAC score, total	21.5 ± 6.1	23.0 ± 5.7	0.221
Preoperative WOMAC pain subscale	13.2 ± 2.8	12.5 ± 3.8	0.312
Postoperative WOMAC pain subscale	4.6 ± 0.8	4.8 ± 1.3	0.372
Preoperative WOMAC stiffness subscale	4.7 ± 1.8	4.3 ± 1.8	0.284
Postoperative WOMAC stiffness subscale	2.5 ± 0.9	2.6 ± 1.0	0.612
Preoperative WOMAC function subscale	31.3 ± 7.2	29.1 ± 8.9	0.191
Postoperative WOMAC function subscale	14.3 ± 4.4	15.8 ± 4.3	0.098
Preoperative FJS	16.2 ± 5.7	14.5 ± 5.3	0.138
Postoperative FJS	58.5 ± 13.4	55.9 ± 14.1	0.362
Preoperative ROM, degrees	114.9 ± 15.8	115.5 ± 14.5	0.848
Flexion contracture, n (%) Recurvatum, n (%) Postoperative ROM, degrees	53.2% 0 125.7 ± 10.1	51.1% 0 123.6 ± 11.4	0.836 0 0.347
Flexion contracture, n (%)	1 (2.1%)	2 (4.3%)	1.00
Recurvatum, n (%)	2 (4.3%)	1 (2.1%)	1.00

KSS, Knee Society Score; WOMAC, Western Ontario and McMaster Universities Osteoarthritis Index; FJS, Forgotten Joint Score; ROM, range of motion; PCO, posterior condylar offset.

**Table 2:** Comparison of radiological outcomes between the two groups at preoperative and 2-year postoperative points. PCO, posterior condylar offset.

	1-mm Group (N = 47)	2-mm Group (N = 47)	*p* Value
Preoperative hip-knee-ankle angle, degrees	Varus 9.7 ± 6.4	Varus 10.2 ± 6.1	0.505
Postoperative hip-knee-ankle axis angle, degrees	Varus 1.0 ± 2.8	Varus 1.0 ± 2.3	0.999
Preoperative PCO, mm	31.6 ± 3.2	30.9 ± 3.5	0.319
Postoperative PCO, mm	35.6 ± 4.2	35.1 ± 4.2	0.565
Preoperative PCO ratio	0.5 ± 0.0	0.5 ± 0.0	0.999
Postoperative PCO ratio	0.5 ± 0.0	0.5 ± 0.0	0.999

**Table 3:** Polyethylene (PE) insert thickness in the two groups.

	1-mm Group (N = 47)	2-mm Group (N = 47)	*p* Value
PE thickness, mm	11.5 ± 1.8	12.8 ± 1.6	0.001
PE thickness, mm (except three patients using 9 mm) Thick (≥13 mm) PE, n (%) Thick (≥14 mm) PE, n (%) Thick (≥15 mm) PE, n (%)	11.6 ± 1.7 11 (23.4%) 6 (12.7%) 3 (6.4%)	12.7 ± 1.8 18 (38.3%) 18 (38.3%) 5 (10.6%)	0.008 0.118 0.005 0.714

## Data Availability

All relevant data generated or analyzed during this study are included in this published article.
